# Heme-Iron-Induced Production of 4-Hydroxynonenal in Intestinal Lumen May Have Extra-Intestinal Consequences Through Protein-Adduct Formation

**DOI:** 10.3390/antiox9121293

**Published:** 2020-12-17

**Authors:** Julia Keller, Sylvie Chevolleau, Maria-Helena Noguer-Meireles, Estelle Pujos-Guillot, Mylène Delosière, Céline Chantelauze, Charlotte Joly, Florence Blas-y-Estrada, Isabelle Jouanin, Denys Durand, Fabrice Pierre, Laurent Debrauwer, Vassilia Theodorou, Françoise Guéraud

**Affiliations:** 1 Toxalim, Research Centre in Food Toxicology, Toulouse University, INRAE UMR 1331, ENVT, INP-Purpan, UPS, F-31300 Toulouse, France; keller.julia@hotmail.fr (J.K.); sylvie.chevolleau@inrae.fr (S.C.); mariahelena.noguer@gmail.com (M.-H.N.-M.); florence.blas-y-estrada@inrae.fr (F.B.-y-E.); isabelle.jouanin@inrae.fr (I.J.); fabrice.pierre@inrae.fr (F.P.); laurent.debrauwer@inrae.fr (L.D.); vassilia.theodorou@inrae.fr (V.T.); 2 Metatoul-AXIOM Platform, National Infrastructure for Metabolomics and Fluxomics, MetaboHUB, Toxalim, INRAE, F-31300 Toulouse, France; 3 Université Clermont Auvergne, INRAE, UNH, Plateforme d’Exploration du Métabolisme, MetaboHUB, Clermont, F-63000 Clermont-Ferrand, France; estelle.pujos@inrae.fr (E.P.-G.); charlotte.joly@inrae.fr (C.J.); 4 Université Clermont Auvergne, INRAE, UMRH, F-63000 Clermont-Ferrand, France; mylene.delosiere@inrae.fr (M.D.); celine.chantelauze@inrae.fr (C.C.); denis.durand@inrae.fr (D.D.)

**Keywords:** heme iron, 4-hydroxynonenal, protein-adducts, lipid peroxidation

## Abstract

Some epidemiological studies show that heme iron consumption, in red meat, is associated to the development of several chronic diseases, including cancers and cardio-metabolic diseases. As heme iron intestinal absorption is finely regulated, we hypothesized that heme iron may act indirectly, through the peroxidation of dietary lipids, in food or in the intestinal lumen during digestion. This heme-iron-induced lipid peroxidation provokes the generation of toxic lipid oxidation products that could be absorbed, such as 4-hydroxynonenal (HNE). In a first experiment, heme iron given to rats by oral gavage together with the linoleic-acid-rich safflower oil induced the formation of HNE in the intestinal lumen. The HNE major urinary metabolite was elevated in the urine of the treated rats, indicating that this compound has been absorbed. In a second experiment, we showed that stable isotope-labeled HNE given orally to rats was able to reach non-intestinal tissues as a bioactive form and to make protein-adducts in heart, liver and skeletal muscle tissues. The presence of HNE-protein adducts in those tissues suggests a putative biological role of diet-originating HNE in extra-intestinal organs. This finding could have major consequences on the onset/development of chronic diseases associated with red meat over-consumption, and more largely to peroxidation-prone food consumption.

## 1. Introduction

Epidemiological studies show that excessive red (but not white) meat consumption has been associated to the onset/progression of several chronic diseases, conclusively for colorectal cancer but also in extra intestinal localizations for other cancers (pancreas, bladder, breast, lung), although in a less obvious way [1]. Red meat consumption is also associated to diabetes, metabolic syndrome and cardiovascular diseases [2]. A particular feature of red meat is its concentration in the pro-oxidant compound heme iron, which makes the red meat red. We have previously shown that this compound may play a central role in colorectal cancer promotion, a digestive disease [3,4,5]. On the other hand, dietary heme iron, but not total or non-heme iron, has been linked to cardiovascular diseases, with a curvilinear association [6]. Heme iron has also been linked to the onset of diabetes [7]. We have previously shown that dietary heme iron, but not ferric citrate given as the same iron molar concentration, could induce a cancer-promoting environment in the colon, through the luminal over-generation of cyto- and geno-toxic alkenals, among which 4-hydroxynonenal (HNE) seems to be the most important [8]. Recently, some authors reported the formation of HNE following heme-induced lipid peroxidation of oil-in-water emulsion, under in vitro gastric digestion conditions [9]. Those lipid peroxidation products may be the second messengers of intestinal heme iron/red meat toxicity. As iron (heme and non heme) absorption is finely regulated [10], we hypothesize that its deleterious effects on non-intestinal tissues may be mediated by the luminal production of those toxic alkenals and their possible action away from their production site, i.e., the digestive tract. We have previously shown that DHN-MA (1,4-dihydroxynonane-mercapturic acid), a major urinary metabolite of HNE [11,12], was elevated in the case of a heme-iron-rich diet in murine models and in humans [13], indicating that HNE was absorbed, metabolized and excreted as less toxic metabolites. In a previous paper, we reported that radiolabeled HNE, given orally to rats, gave metabolites in all the tested tissues. However, it was not possible at the time to identify those tissue metabolites and more precisely, to show that unmetabolized reactive HNE was able to reach non-intestinal tissues [14]. In the present communication, we showed that an orally given mixture of heme iron and omega-6-rich dietary oil induced the intestinal luminal formation of HNE. In a separate experiment, we also showed that orally given stable isotope-labeled HNE was able to reach non intestinal tissues as a bioactive form and to make protein-adducts in those tissues such as heart, liver and skeletal muscle. HNE-protein adducts of endogenous origin, i.e., formed upon cellular lipid peroxidation/oxidative stress, have been extensively studied in the literature [15]. Those compounds are involved in the pathogenesis of numerous chronic diseases linked to inflammation processes [16,17]. Their implication as a cause or a consequence is, however, still a matter of debate. However, to the best of our knowledge, this is the first report of extra-intestinal HNE-protein adducts specifically formed upon oral administration. This finding could have major consequences on the onset/development of chronic diseases associated with red/processed meat over-consumption, and more largely to peroxidation-prone food consumption. 

## 2. Materials and Methods 

Chemicals: Methanol (Optima LC/MS grade) and acetonitrile (Optima LC/MS grade) were purchased from Fisher (Illkirch, France), formic acid from Sigma Aldrich (St Quentin Fallavier, France). Ultra-pure water was obtained using a Milli-Q system (Millipore, St Quentin en Yvelines, France). 1-((ammoniooxy)methyl)-2-bromobenzene chloride (BBHA) was purchased from Interchim (Montluçon, France). Piperazine-*N*,*N*′-bis(2-ethanesulfonic acid) (PIPES) and trifluoroacetic acid (TFA) were purchased from Acros organics (Geel, Belgium).

[1,2-^13^C_2_]-4-Hydroxy-2(*E*)-nonenal (^13^C-HNE). ^13^C-HNE was prepared according to our published method [18], with modifications. Ethyl [1,2-^13^C_2_]-2-bromoacetate was converted to ethyl 4-hydroxy-2(*E*)-nonenoate by reacting consecutively with 4-chlorothiophenol, hydrogen peroxide, and *n*-heptanal as described [19]. The remaining steps were unchanged.

4-Hydroxy-2(*E*)-nonenal (HNE). HNE was prepared from ethyl 2-bromoacetate by the same method as for ^13^C-HNE.

### 2.1. Animals

HNE neoformation experiment: Male Fischer 344 rats (100 g) were purchased from Charles River (France). Animal care was in accordance with our local ethic committee (APAFIS#6350). Rats were given by gavage a freshly prepared mixture of hemin and safflower oil (8 rats), or only safflower oil (4 rats) and placed in plastic metabolic cages to collect separately urine and feces for 24 h. They were given tap water and commercial rat show ad libitum during this period. The quantities of hemin and safflower oil represented the amount given for a day in our previous experiments on hemin promotive effects on colorectal cancer [5,8], namely 18.8 mg of hemin in 1 g of safflower oil, corresponding to the quantities present in 20 g of a diet containing 5% safflower oil and 0.094% hemin. Two hemin treated rats and one control rat were sacrificed by progressive CO_2_ inhalation at the different time points of the experiment: 1, 4, 8 and 24 h after the gavage. The digestive tract was removed and the stomach and intestinal contents collected and kept at −80 °C until analysis. Fecal waters were prepared as follows: feces samples were weighed and a double amount of distilled water containing 5% 0.45 M butylated hydroxytoluene (BHT) was added. The mixture was crushed with ceramic beads using Fast-Prep (MP Biomedicals, France). After centrifugation at 5500 *g* for 20 min, the supernatant (“fecal water”) was collected and kept at −80 °C until use.

^13^C-HNE experiment: Male Wistar rats (200 g) were purchased from Charles River (France). Animal care was in accordance with our local ethic committee (number TOXCOM/0008/FG). A total of 3 rats were treated by gavage with 1 mL of an equimolar mixture of ^12^C-HNE and ^13^C-HNE diluted in water (1 mg of each HNE isotope). HNE concentration was checked spectrophotometrically (λ, 223 nm; ε, 13,750 L/mol/cm). The rats were housed in individual metabolism cages and a commercial rat show and tap water were provided ad libitum. A total of 24 h after HNE exposure, rats were sacrificed by progressive CO_2_ inhalation; tissues were collected and stored at −80 °C until further analysis.

### 2.2. Assays

Luminal and fecal HNE/HHE: Luminal and fecal HNE and 4-hydroxyhexenal (HHE) were measured by ultra-high-pressure liquid chromatography tandem mass spectrometry with electrospray ionization (UHPLC-ESI-MS/MS) according to a previously developed method for HNE and HHE quantification [20], validated according to the EMEA (European MEdicines Agency) guidelines [21], for selectivity, sensitivity, linearity, carry-over effect, recovery, matrix effect, repeatability, trueness and intermediate precision. Briefly, luminal and fecal water samples were derivatized in situ using a brominated reagent (BBHA) in the presence of deuterated internal standard (HNE-^2^H_11_/HHE-^2^H_5_), extracted by solid phase extraction (SPE), and then analyzed by LC-positive ESI-MS/MS in multiple-reaction monitoring (MRM) mode. The UHPLC-MS/MS analyses were performed on a TSQ Vantage triple-stage quadrupole mass spectrometer (Thermo Scientific, Les Ulis, France) equipped with an Accela 600 pump (Thermo Scientific). The column used was a Hypersil Gold C18 (50 × 2.1 mm, 1.9 µm) from Thermo Scientific, with 1.9 μm prefilter and heated at 40 °C. Chromatographic separation was achieved using mobile phases composed of A: H_2_O/CH_3_CN 85/15 + 0.1% formic acid and B: CH_3_CN/H_2_O 95/5 + 0.1% formic acid, with a flow rate of 0.3 mL/min. Initial conditions were A:B 100:0 *v/v* held for 1 min, followed by a 15 min linear gradient from A:B 100:0 *v/v* to A:B 0:100 *v/v*, then held for 2 min. Typical ionization working parameters were as follows: nebulizer temperature, 420 °C; heated transfer capillary temperature, 320 °C; transfer capillary voltage, 4800 V; sheath gas flow rate, 25 a.u.; auxiliary gas flow rate, 15 a.u. sweep gas flow rate, 3 a.u. For each molecule, the signal was monitored on the basis of the chromatographic retention time of the BBHA derivative (two Z and E configuration isomers), and of the MRM transitions for each brominated derivative. Quantification and confirmation transitions were monitored for the two hydroxy-alkenals (namely *m/z* 340 > 169 and *m/z* 340 > 224 for HNE-BBHA and *m/z* 298 > 169 and *m/z* 298 > 224 for HHE-BBHA) and their respective internal standard (*m/z* 351 > 169 and *m/z* 351 > 225 for HNE^2^H_11_-BBHA and *m/z* 303 > 169 and *m/z* 303 > 227 for HHE^2^H_5_-BBHA) at their corresponding optimal collision energies. 

Urinary and fecal DHN-MA: Urinary and fecal DHN-MA were measured by competitive Enzyme Immuno Assay (EIA) as previously described [22] for urine samples, using a home-made anti-DHN-MA polyclonal antibody, Bertin Bioreagent DHN-MA-AChE (AcetylCholine Esterase) tracer and Ellman’s reagent (Montigny-le-Bretonneux, France). Dilution ranges for urine were 100–10,000 and 20–2000 for fecal waters.

Fecal HNE-protein adducts: Fecal HNE-protein adducts were measured using indirect competitive ELISA assay as described by Satoh et al. [23], with slight modifications. Briefly, HNE-casein adducts were prepared by mixing 3.2 mg of pure HNE to 8 mL of 1 mg/mL casein in 50 mM phosphate buffer pH 7.4 and stirring the solution overnight. 96-well plates (Nunc Maxisorp) were coated overnight with 3.75 µg HNE-casein/mL in carbonate buffer (100 µL), washed and then blocked 1 h with phosphate-buffered saline (PBS) containing 0.5 mg/mL casein (PBS-casein buffer). Standards (HNE-casein) or samples (60 µL) were mixed in a separate plate with an equal volume of monoclonal anti-HNE-histidine antibody (1/1000 in PBS-casein buffer) [24,25]. After 30 min, this mixture was transferred to the coated/washed/blocked/emptied microplate and left for competition between the coated HNE-casein adducts and the sample/standard HNE-protein adducts for 1 h, under mild agitation at room temperature. After a washing step, secondary antibody (Goat anti-mouse horseradish peroxidase (HRP)-conjugated antibody (ImmunoReagents Inc, Raleigh, NC, USA), 1/2000 in PBS-casein buffer) was added to the plate for 1 more hour. The plate was then washed again, 100 µL TMB (3,3′, 5,5;-tetramethylbenzidine) reagent (Sigma-Aldrich) was added to the wells and left until the color developed properly, then the reaction was stopped with 50 µL 2N H_2_SO_4_. The absorbance was read at 450 nm using a microplate reader. Fecal water dilution range was 10–100 in PBS-casein buffer.

Tissue HNE-protein adducts detection: tissues (heart, liver and hind leg muscle) from [^13^C-HNE/^12^C-HNE 50/50] treated rats were prepared as follows:

Each tissue was finely grounded in liquid nitrogen to limit oxidation and stored at −80 °C. HNE-protein adducts were labelled and reduced in [^2^H_1_]DHN-protein adducts (primary alcohols) by NaB[^2^H_4_] (0.2 mmol) in ethylenediaminetetraacetic acid (EDTA) (50 mM)/Hepes (2 mM) buffer to stabilize them. Proteins were precipitated by saturated sulfosalicylic acid solution. After centrifugation at 4 °C and 6500 rpm for 10 min, the supernatants were resuspended by sonication in a guanidine (8 M)/EDTA (13 mM)/Tris (133 mM) buffer pH7.1. Then, 0.8 nmol of [^2^H_11_]DHN was added as an internal standard and the protein bonds were cleaved in the presence of Raney nickel by a 14h incubation at 55 °C. The compounds were extracted with ethyl acetate, dried with Na_2_SO_4_ and filtered. The dried residues were silylated with *N*-*tert*-Butyldimethylsilyl-*N*-methyltrifluoroacetamide (MTBSTFA) + 1% *tert*-butyldimethylchlorosilane (TBDMCS). Samples were then analyzed using a triple quadrupole mass spectrometer (MS) Quattro Micro (Waters Corporation, Manchester, UK) coupled with a Gas Chromatograph (GC) Agilent 6890N system (Agilent Technologies, Palo Alto, CA, USA), operating in positive chemical ionization mode using isobutane as reagent gas. Injections (1 µL) were made at 250 °C using split mode (1/20). High-purity helium was used as carrier gas, at a constant flow rate of 0.7 mL/min. Chromatographic separation was performed using an HP-5MS Agilent Technologies capillary column (50 m × 0.2 mm diameter, 0.33 µm film thickness) under the following conditions: 170 °C for 1 min, increased by 10 °C/min until 220 °C, 2 °C/min until 235 °C, 5 °C/min until 250 °C and then by 30 °C/min until 300 °C. At the end of each run, temperature was kept at 300 °C for 5 min to clean the column. MS parameters were optimized using standard solutions. Maximum sensitivity was obtained for an ion-source temperature set at 120 °C, an electron energy at 90 eV and an emission current at 200 mA. Quantification was achieved by measuring product ions (multiple reaction monitoring) from the fragmentation of the protonated [M + H]^+^ molecules. Collision energy potentials were then adjusted to optimize the signal for the most abundant product (daughter) ions: *m/z* 389 > 257 for DHN (endogenous ^12^C-DHN), *m/z* 390 > 258 for [^2^H_1_]DHN (reduced ^12^C-HNE) and *m/z* 392 > 260 M+2 [^2^H_1_]DHN ^13^C (reduced ^13^C-HNE) using argon as collision gas. Quantities of [M+2]DHN ^13^C protein adducts were determined using corrected areas by quantities of endogenous DHN. [^2^H_11_]DHN was used as internal standard for control quality of the analytical system stability.

### 2.3. Statistical Analyses

**Figure 1:**
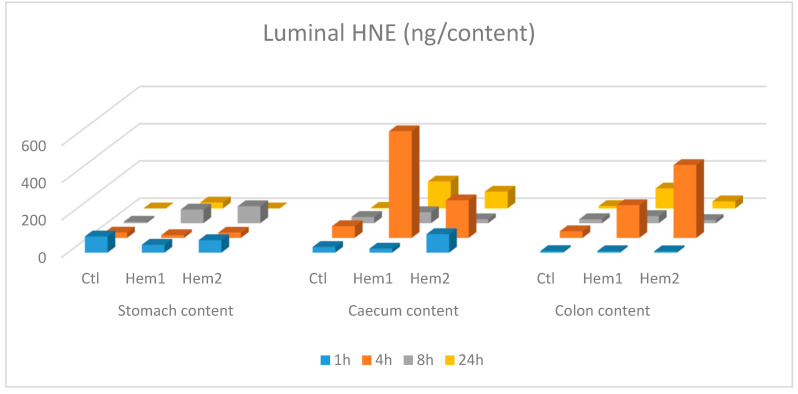
Amounts of 4-hydroxynonenal (HNE) (ng/content) measured in 3 intestinal contents, at 4 different time points, after oral gavage of safflower oil +/− hemin. For each time point, 3 animals were sacrificed: Ctl had received only safflower oil at the starting time point (T0), while Hem1 and Hem2 had received a mixture of safflower oil + hemin at T0. The bars represent the amount of HNE in the intestinal content of one rat, at a specific time point.

**Figure 2:**
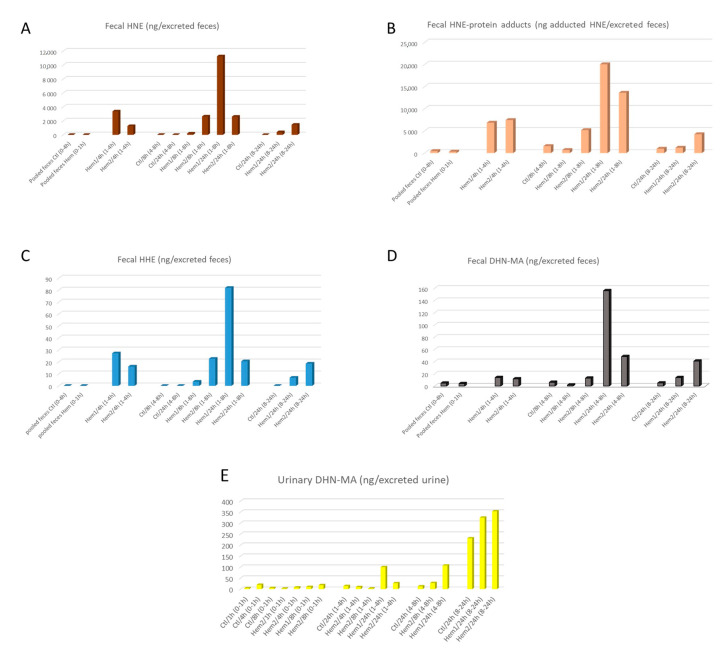
Excretion of fecal HNE, 4-hydroxyhexenal (HHE) and HNE metabolites in feces and urine after oral gavage of safflower oil +/− hemin. (**A**) fecal HNE; (**B**) fecal HNE-protein; (**C**) fecal HHE; (**D**) fecal 1,4-dihydroxynonane-mercapturic acid (DHN-MA); (**E**) Urinary DHN-MA. Samples are identified as “treatment/time of sacrifice (sampling period)” with Treatment = “Ctl”, “Hem1” or “Hem2”; time of sacrifice = 1, 4, 8 or 24 h; feces from different animals were pooled when samples were too small: Ctl feces from 0 to 4 h and Hem feces from 0 to 1 h. For each time point, 3 animals were sacrificed: Ctl had received only safflower oil at the starting time point (T0), while Hem1 and Hem2 had received a mixture of safflower oil + hemin at T0. Each bar represents the excretion of one animal, except when indicated “pooled feces”.

Because feces excretion vary depending on the individuals during the various sampling times, we analyzed, for statistical purpose, data of HNE, HNE-protein adducts and DHN-MA concentration in fecal waters, instead of amounts per contents as shown in Figure 1 and Figure 2. Two-way Analysis Of Variance (ANOVA) was performed (using a Log transformation when necessary) with sampling time and hemin treatment as factors, using GraphPad Prism software.

## 3. Results and Discussion

When rats were given heme iron together with safflower oil (containing mainly omega-6 fatty acids as linoleic acid) by gavage, HNE was found in important amounts in their caecum and colon content as soon as 4 h after (Figure 1), and in their feces 8 h after (Figure 2A). Protein-adducted HNE was found in feces too, with amounts matching those of free HNE (Figure 2B), and in the same order of magnitude (10–20 µg/excreted feces/sampling period 1 to 8 h post gavage, for hemin treated rats vs. less than 2 µg for rats given only safflower oil). HNE was also present in a moderate concentration in the stomach, just after (1 h) the gavage, but there was no marked difference between heme iron fed rats and control rat, fed only safflower oil (Figure 1). When two-way ANOVA (sampling time and hemin treatment as factors) was performed on HNE and HNE-protein fecal water concentrations, we observed that hemin significantly increased the fecal water concentration of HNE (*p* < 0.0001 for hemin treatment) and of HNE-protein adducts (*p* = 0.027 for hemin treatment), when considering data for 4, 8 and 24 h sampling times. HNE was not found in small intestine contents, except for two hemin treated rats, with an amount of 20.9 ng in the proximal part of the intestine (namely the duodenum and the half of the jejunum), one hour after gavage and an amount of 31.5 ng in the inferior part of the intestine (namely the second half of the jejunum and the ileum), four hours after gavage. Interestingly, DHN-MA, a major metabolite of HNE, usually found excreted in urine, was also found in feces with excretion profiles roughly matching the HNE fecal excretion (Figure 2D), but, however, with 10-fold lower concentrations. This hemin-induced DHN-MA excretion increase in fecal water was, however, not statistically significant (*p* = 0.09). This compound was also found in the intestine contents, including the upper tract, indicating that intestinal cells, rather than intestinal flora, were able to metabolize and excrete HNE metabolites in the intestinal lumen. We found up to 5.73 ng of DHN-MA in the proximal part of the intestine and up to 3.84 ng in the distal part, in hemin treated rats, one hour and four hours after gavage, respectively. Amounts in the stomach content were below 1 ng.

Altogether, those results show that free HNE and protein-adducted HNE were formed in the intestinal lumen after heme iron and omega-6-rich oil consumption. The fact that important amounts were found particularly in the distal parts (caecum and colon) could be due to a slowing down of transit in these “reservoir” organs when compared to small intestine. In the small intestine, we probably missed the presence of HNE in half of the rats sacrificed at 1 and 4 h, probably due to a different transit time between animals. The presence of free reactive HNE could have deleterious consequences, as HNE is a cytotoxic and genotoxic lipid peroxidation product. 

On the other hand, HHE that is formed upon omega-3 fatty acid peroxidation, was not found in the digestive tract and only in very limited amounts in the fecal waters of hemin treated rats (Figure 2C), with a mean concentration of 25 ng/mL. This could be associated to an inflammatory process associated to heme iron and polyunsaturated fatty acid consumption [8,26] as peroxidation of dietary fatty acids could not be invoked, due to the absence of omega-3 fatty acid in safflower oil. 

As in our previous studies [5,8], we observed in the present one an increased urinary excretion of DHN-MA in hemin treated rats, when compared to control ones (Figure 2E). However, the amounts excreted are quite moderate when compared to those previous studies. This can be explained by the fact that the urines were collected only during 24 h post gavage, likely reflecting an uncompleted absorption/metabolization process.

In a previous work [14], we reported that radiolabeled HNE, given orally by gavage to rats, was excreted in a large extent as less active/toxic metabolites into urine, indicating that this orally given HNE can be adsorbed during digestion. We also reported that radioactivity was present in all the rat organs. This radioactivity could be attributed to HNE metabolites, HNE-protein adducts or tritiated water due to complete HNE metabolization, and less likely to free HNE as this compound is reactive. At this time, using global liquid scintillation counting after tissue combustion, we were not able to distinguish between those products in the rat tissues. Yet, we believe that it is essential to know whether this radioactivity may correspond, at least in part, to the formation of HNE-protein adducts. This would indicate that HNE could reach all tissues in an active form, and that, consequently, dietary HNE (or HNE formed during digestion in the intestinal lumen) would have biological and even toxic consequences away from the digestive tract. 

In the present work, using a stable isotope labeled-HNE, we showed that orally given HNE is able to make protein adducts in non-intestinal tissues, namely liver, heart and hind leg muscle (Table 1). The use of isotopic labeling enabled us to be sure that the HNE-protein adducts observed were originating from the orally given HNE, as HNE is also an endogenous product, formed upon cellular lipid peroxidation. The proportion of tissue HNE-protein adducts originating from orally given HNE was calculated as twice the proportion of ^13^C-labeled-HNE-protein adducts, as rats were given an equimolar mixture of labeled and unlabeled HNE. This proportion reached 25% of total HNE-protein adducts in the heart, and between 15 and 20% for liver and muscle. This represents significant amounts of adducts that could have important biological consequences.

In the literature, HNE protein adducts were reported to be involved, either as causes or consequences, in numerous chronic diseases, including inflammatory and neurodegenerative ones [27,28]. In those studies, it is unfortunately not possible to distinguish between HNE-protein adducts of endogenous (due to inflammation) and dietary origin. On the other side, some groups reported toxicity of orally given HNE or related compounds. One study on the effect of a single dose of orally given HNE reported a hepato- and nephro-toxicity, with doses ranging from 10 to 1000 mg/kg BW [29], the smaller dosage corresponding to the one used in our present study. These authors observed dose-dependent liver necrosis 14 days after treatment. The same group had reported a liver tumorigenic effect of crotonaldehyde, another alpha-beta-unsaturated aldehyde, given chronically in drinking water [30]. Kang et al. [31] also reported an alteration of hepato- and nephro-toxicity biomarkers in rats given HNE orally each day for 28 days, at doses ranging from 0.5 to 12.5 mg/kg BW/day. A group reported that oxidized products of linoleic acid, a precursor of HNE, induced non-intestinal toxic effects, and particularly a fatty infiltration of the liver [32]. The same group gave orally to rats a low molecular weight fraction of autoxidized radiolabeled linoleic acid metabolites, containing HNE. They report the presence of radioactivity in extra-intestinal tissues 6 h after, especially in liver, kidney, brain, heart and lung [33]. This parallels our results obtained with radiolabeled HNE [14]. Another group reported an increase in HNE in the liver 15 h after an intra-gastric dose of linoleic acid hydroperoxides [34]. The same group reported hepatotoxicity caused by those dietary secondary products of lipid peroxidation [35]. All these studies strongly suggest that dietary HNE, and related compounds, may reach extra-intestinal tissues and have deleterious consequences. The present work provides the proof of this assumption, at least for the tissues we tested.

The aim of this communication was to confirm, using a restricted number of animals, the neoformation of HNE in the intestinal lumen following an oral exposure to heme iron and an omega-6-fatty-acid-rich oil and the presence of diet-originating HNE-protein adducts in extra-intestinal organs. Our pioneering results with the stable isotope-labeled HNE pave the way for further studies to determine whether foods that cause luminal lipid peroxidation, such as red meat, may be implicated in various chronic diseases, and the putative role of HNE and other alkenals in this implication.

## Figures and Tables

**Table 1:** HNE-protein adducts from orally given HNE: Semi-quantitative evaluation of ^13^C-labeled-HNE-protein adducts: % of labeled HNE-protein adducts among total HNE-protein adducts. As rats were given an equimolar mixture of labeled and unlabeled HNE, the percentage of HNE-protein adducts from orally given HNE is quantified as twice the percentage of ^13^C-labeled-HNE-protein adducts. The results are the mean of n animals.

	% of [^13^C-HNE]-Protein Adducts	n	% of HNE-Protein Adducts from Orally Given HNE
Muscle	7.9	1	15.8
Liver	9.3	3	18.6
Heart	12.3	3	24.6
